# Identification and DNA annotation of a plasmid isolated from *Chromobacterium violaceum*

**DOI:** 10.1038/s41598-018-23708-5

**Published:** 2018-03-28

**Authors:** Daniel C. Lima, Lena K. Nyberg, Fredrik Westerlund, Silvia R. Batistuzzo de Medeiros

**Affiliations:** 1 Instituto Federal de Educação, Ciência e Tecnologia do Rio Grande do Norte, Natal, Brazil; 2 Laboratório de Biologia Molecular e Genômica, Universidade Federal do Rio Grande do Norte, Natal, Brazil; 3 Department of Biology and Biological Engineering, Chalmers University of Technology, Gothenburg, Sweden

## Abstract

*Chromobacterium violaceum* is a ß-proteobacterium found widely worldwide with important biotechnological properties and is associated to lethal sepsis in immune-depressed individuals. In this work, we report the discover, complete sequence and annotation of a plasmid detected in *C. violaceum* that has been unnoticed until now. We used DNA single-molecule analysis to confirm that the episome found was a circular molecule and then proceeded with NGS sequencing. After DNA annotation, we found that this extra-chromosomal DNA is probably a defective bacteriophage of approximately 44 kilobases, with 39 ORFs comprising, mostly hypothetical proteins. We also found DNA sequences that ensure proper plasmid replication and partitioning as well as a toxin addiction system. This report sheds light on the biology of this important species, helping us to understand the mechanisms by which *C. violaceum* endures to several harsh conditions. This discovery could also be a first step in the development of a DNA manipulation tool in this bacterium.

## Introduction

*Chromobacterium violaceum* is a Gram-negative facultative anaerobe bacillus belonging to the *Neisseriaceae* family^1^. This free-living ß-proteobacterium reside mainly around tropical and sub-tropical regions. The study of *C. violaceum* started in the 1970s, focusing on its potential in pharmacology and industry for the production of antibiotics, anti-tumoral substances, biopolymers and others organic compounds (reviewed in refs^2–4^). *C. violaceum* is also an opportunistic pathogen that can cause severe infections and lead to sepsis and sometimes death in immuno-depressed individuals^5,6^.

In 2003, the complete genome of *C. violaceum* was sequenced and many genes related to stress adaptability were identified. This led to a great number of studies of how the bacterium copes with environmental challenges^7–11^. Many studies focusing on understanding the mechanisms of *quorum sensing* in *C. violaceum* make this organism an important model species^12–14^.

Despite the great interest in *C. violaceum* and the sequencing of its entire genome^7,8,15^, efficient methods to modify its genome are still not developed. For example, a study reported genetic transformation of *C. violaceum*^16^ but this methodology proved to be irreproducible by many groups. More recently, a group succeeded in generating mutants in *C. violaceum* using conjugation^17^. This method is laborious and mutants often revert. Therefore, there is a demand to develop more efficient tools to conduct genetic studies in *C. violaceum*.

Here, we used single DNA molecule analysis and next-generation sequencing to identify a plasmid in *C. violaceum* strain ATCC 12472. The presence of this 44,212 bp plasmid has been unnoticed until now and its characterization may help building a shuttle vector that would greatly facilitate the development of genome engineering tools for *C. violaceum*.

## Experimental Procedures

### Plasmid isolation

Four isolated colonies of *C. violaceum* ATCC 12472 were inoculated in four flasks containing 400 mL of LB medium each for 16–18 h. The cultures were centrifuged at 4 °C, 5 minutes, 7441 × *g*. The pellets were resuspended with 25 mL of Ressuspension Buffer (50 mM Tris-HCl, 10 mM EDTA, RNAse 100 μg/mL, pH 8.0), and then 25 mL of Lysis Buffer (SDS 1%; 0.2 M NaOH) was added, with 5 minutes of room temperature incubation. The plasmid DNA was precipitated by adding 25 mL of 3 M Potassium Acetate, pH 5.5, followed by centrifugation at 22789 × *g*, 10 minutes at 4 °C. The supernatant was transferred to a new tube and 0.7 volume of isopropanol was added. After one more step of centrifugation (22789 × *g*, 10 minutes, 4 °C), the pellets were re-suspended with 1 mL of TE Buffer and one volume of phenol:chloroform was added. After centrifugation (22789 × *g*, 10 minutes, 4 °C), the aqueous phase was transferred to a new tube and the DNA was precipitated with one volume of isopropanol. Finally, the DNA was washed with 80% Ethanol and the four independent preparations were re-suspended with 500 μL of TE.

In order to certify that our preparation was free of genomic DNA we isolated the band containing the plasmid and digested the agarose using ß-agarase (NEB catalog # - M0392S) according to the manufacturer’s instructions.

### Optical analysis of DNA in nanochannels

The optical DNA mapping of the single plasmid molecules were performed as described in ref.^18^. Using a combination of a DNA fluorescent dye (YOYO-1) and Netropsin, an antibiotic that binds specifically to AT DNA regions, this technique allows the acquisition of DNA barcode images, with dark and bright regions corresponding to AT-rich and GC-rich regions respectively^19^. In this way, the pattern of the emission intensity reflects the sequence of the DNA molecule, with a resolution on the kilobasepair length scale. The nanofluidic chips were fabricated in fused silica, using conventional techniques, as described in ref.^20^. All the data was recorded, using a Zeiss AxioObserver. Z1 microscope equipped with a 100× TIRF oil immersion objective (NA = 1.46) from Zeiss and a Photometrics Evolve EMCCD camera.

### NGS Sequencing and Assembly

The DNA was quantified using Qubit Fluorometric Quantitation and the quality was checked on an agarose gel. The library was prepared using TruSeq Nano DNA Sample Preparation Kit (Illumina) according to the manufacturer’s instructions and then sequenced on the Illumina MiSeq at Fasteris SA. For the base-calling, the CASAVA pipeline 1.8 was used. *De novo* genomic assembly was made using VELVET v1.2.10 and Burrows-Wheeler Alignment Tool (v0.5.9) for mapping.

### Plasmid annotation and comparison

The annotation was made using Glimmer (v3.02b), a software built to find genes in bacteria, archaea and viruses. Bacteria/archaea genetic code and circular topology were chosen. The search for homology of the whole pChV1 sequence was made using the BLASTn program against non-redundant (NR) NCBI database and against a specific bacteriophage database (unclassified bacteriophages – taxid: 12333), also from NCBI. Comparison of the predicted ORFs in genomic databases was made using BLASTx. Hits with more than 50% coverage and with the highest BitScore were picked. Search for tRNAs was made using the online version of tRNAscan-SE v1.21 in default mode. DNA inverted repeated sequences were obtained using Einverted (http://emboss.bioinformatics.nl/cgi-bin/emboss/einverted). The search for palindromic DNA was made using the MEME web-tool^21^. GC content profile and GC-skew were obtained using GC-Profile^22^ and GenSkew (http://genskew.csb.univie.ac.at/), respectively.

### Data availability

The *pChV1* complete sequence is available at GenBank (accession number - MG651603). FASTQ file is also available in the Sequence Read Archive (SRA) repository with accession number SRR6363036.

## Results

### Identification of an episome in *C. Violaceum* strain ATCC 1242

**Figure 1:**
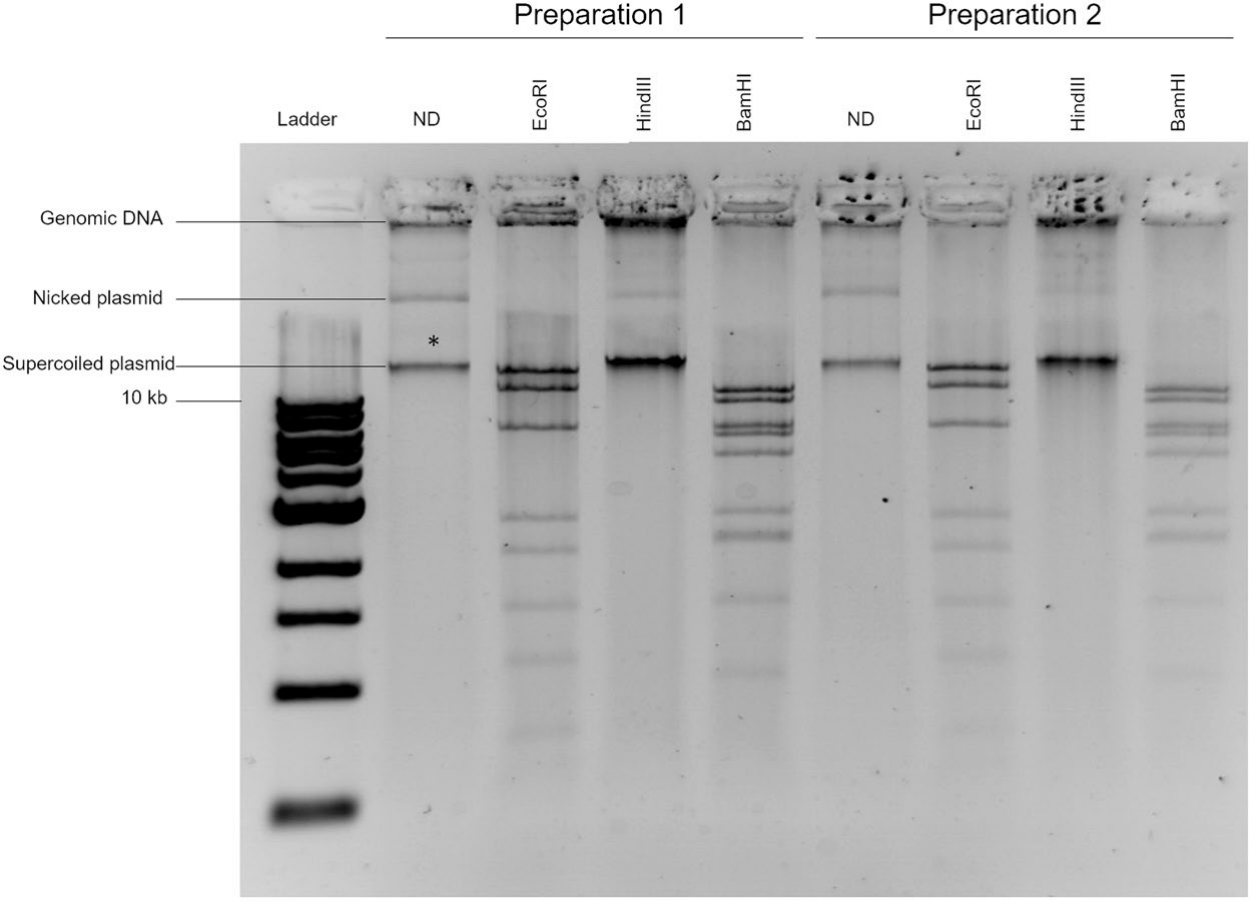
Restriction digestion pattern from two independent preparations of the episome. The asterisk denotes the band corresponding to the plasmid. The restriction enzyme used is mentioned on top of each lane. 0.8% TBE Agarose gel stained with ethidium bromide.

**Figure 2:**
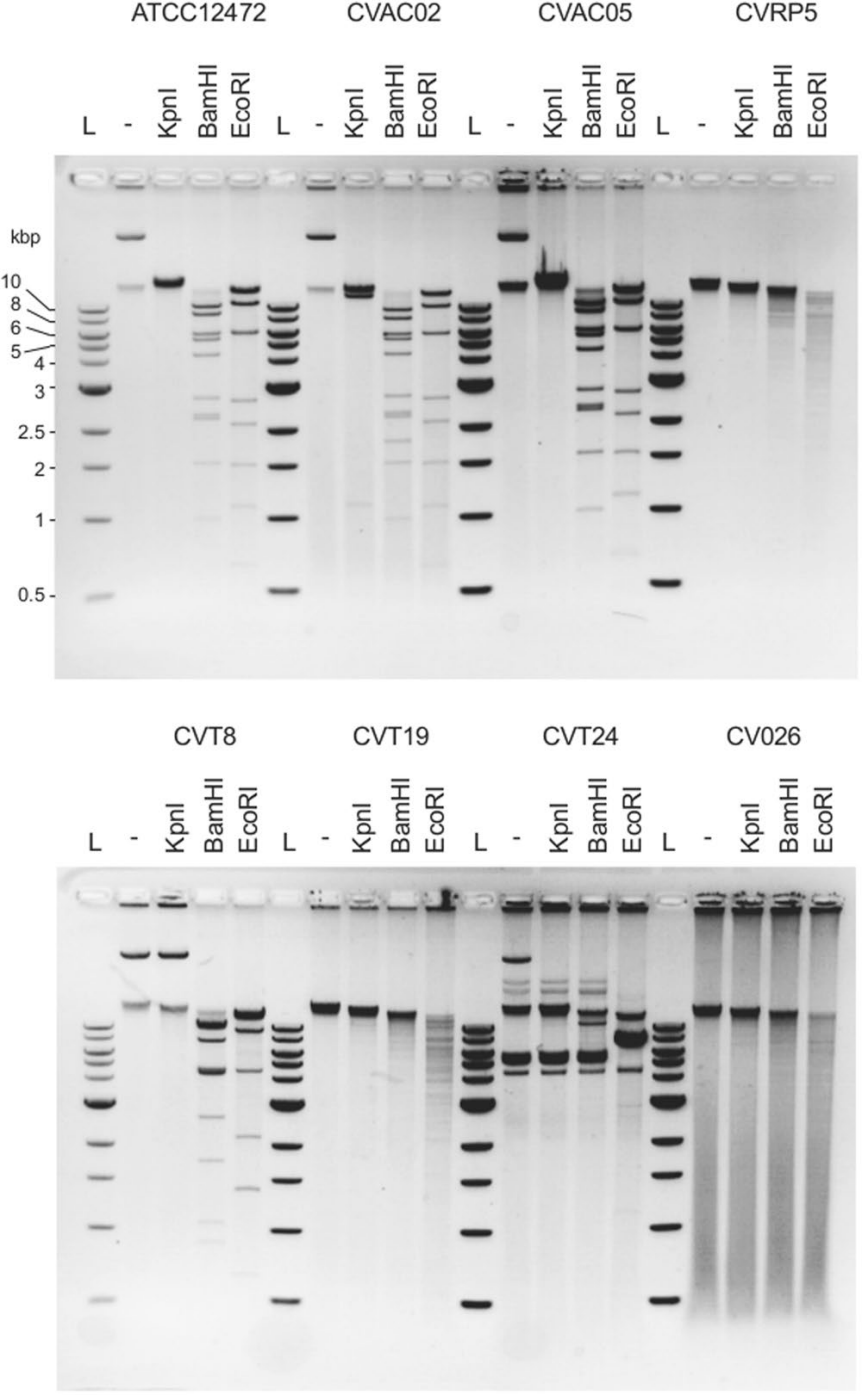
Restriction digestion of extra-chromosomal DNA extracted from eight different *C. violaceum* strains. Samples were digested with KpnI, BamHI and EcoRI for one hour at 37 °C. “−” reflects non digested samples and L is DNA ladder. 0.8% TBE agarose gel stained with ethidium bromide.

While extracting genomic DNA from *C. violaceum* strain ATCC 12472 to construct a genomic library, we noticed after agarose gel electrophoresis the recurrence of a DNA species smaller than expected for high molecular weight genomic DNA in our preparations. We hypothesized that this DNA species could be a circular episome. We therefore carried out standard plasmid DNA preparations and analyzed the purified DNA by agarose gel electrophoresis and ethidium bromide staining. As can be seen in lane 2 of Fig. 1, the preparation contained contaminating high molecular weight genomic DNA trapped in the well but also a species with mobility much greater than 10 kb, our putative episome as indicated by a star symbol. A third faster migrating species was also observed. We next performed a restriction enzyme analysis of our preparation with KpnI, BamHI or EcoRI (Fig. 1 lanes 3–5). Consistent with linearization of a circular DNA molecule, digestion with KpnI resulted in a single band and disappearance of genomic DNA, both the DNA trapped in the well and the third species described above, due to characteristic smearing of genomic DNA digestions (Fig. 1 lane 3). Digestion with BamHI or EcoRI resulted instead in defined patterns of discrete DNA fragments (Fig. 1 lanes 4 and 5). We then performed the same analysis using seven different additional *C. violaceum* strains (Fig. 2). Preparations from strains CVAC02, CVAC05, and CVT8 appeared to contain a putative episome similar to strain ATCC 12472, while preparations from strains CV026, CVT19 and CVRP5 appeared to contain only genomic DNA. The preparation from strain CVT24 also seemed to contain a putative episome species but the result from the restriction analysis is difficult to interpret. Thus, we identified an episome in *C. violaceum* strain ATCC 12472 and propose to name it pChV1.

### Episome pChV1 is a circular plasmid

**Figure 3:**
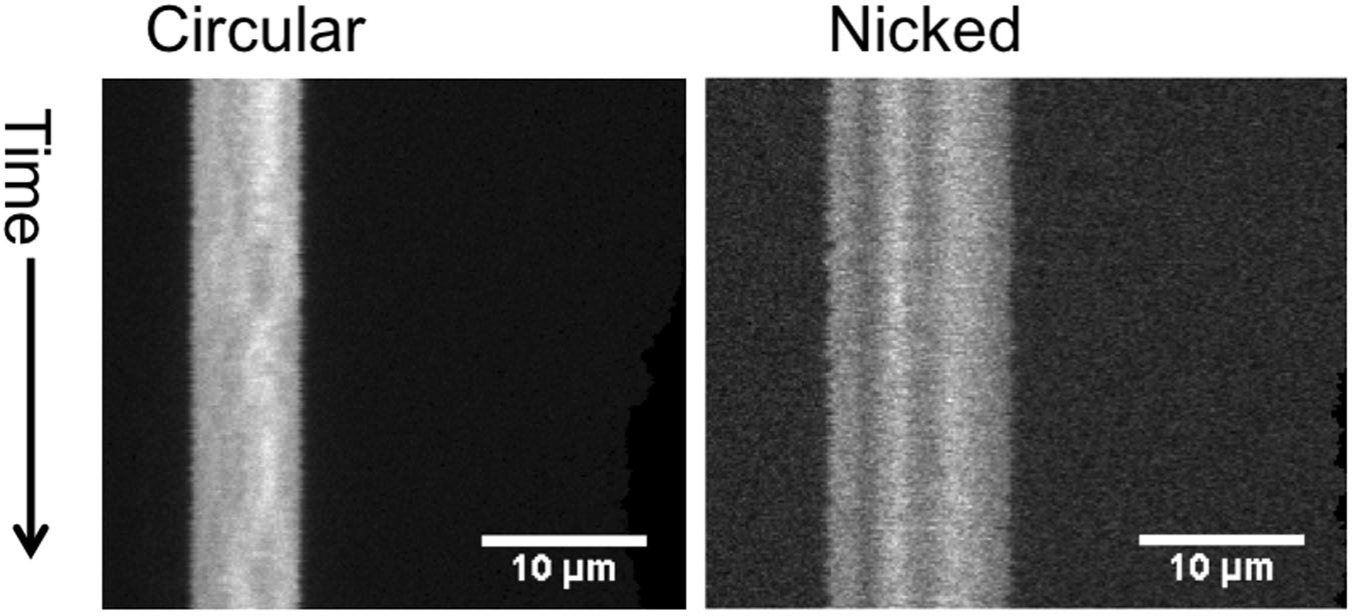
Kymographs showing the extensions of circular and nicked forms of the episome in nanofluidic channels. Competitive binding was used in order to produce the emission intensity pattern along the linear for of the plasmid.

The above restriction enzyme analysis suggested that pChV1 is a circular DNA molecule. To verify this hypothesis the episome DNA was purified after gel electrophoresis (Fig. 1 lane 2, band indicated with a star symbol) and stained with YOYO-1 (a bis-intercalator fluorescent dye) and Netropsin (a minor groove binder of AT-rich sequences of double-stranded DNA) that competes with YOYO-1 intercalation^19^. Such stained preparations were diluted and injected in nanochannels to observe individual extended episome DNA molecules by fluorescence microscopy (Fig. 3A). Initially, the contour length of individual episome DNA molecules averaged circa 6 μm (Fig. 3A - left kymograph). However, after prolonged illumination, the accumulation of nicks in the DNA molecule induced double strand breakage and linearization of the circular plasmid, evidenced by an increase in contour length (Fig. 3B - right kymograph). Lambda phage DNA (48,502 bp) was used as an internal standard to convert extension from pixels to basepairs. Using a scaling factor of 1.84^23^ for going from circular to linear extension, we estimated that the longest episome molecule detected was circa 44 kb in size. DNA barcode analysis revealed mostly GC-rich regions with two AT-rich regions of darker signal.

### The pChV1 DNA sequence

**Figure 4:**
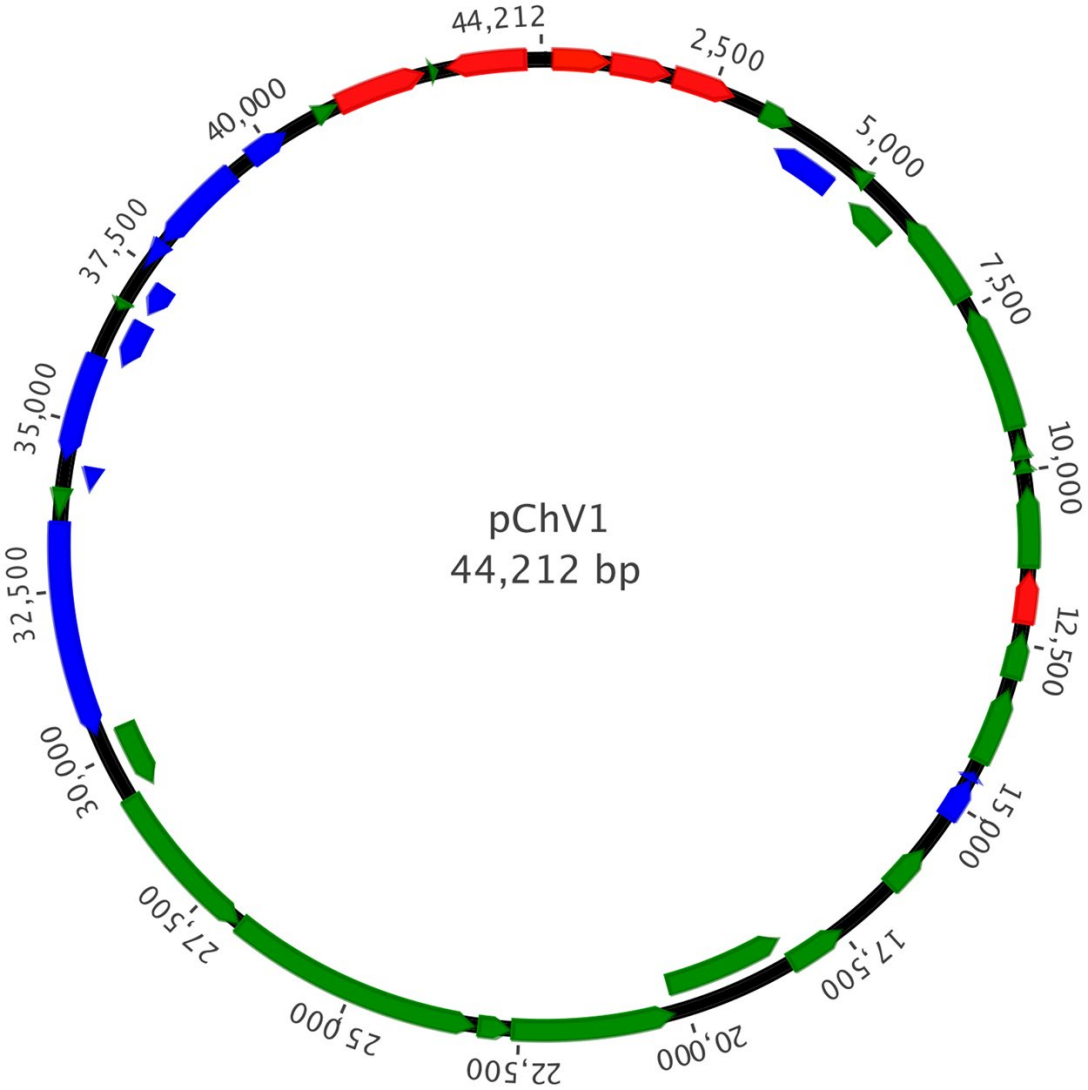
Map of the pChV1 plasmid. Most of the phage-related genes are present in the same region. The distribution of the plasmid partitioning genes is in accordance to what is seen in the literature. Blue, red and green arrows depict phage, plasmid and hypothetical ORFS respectively.

The complete sequence of pChV1 revealed a circular element with 44,212 bp with a G + C content of 65.96% (Table 1). 39 Open Reading Frames (ORFs) were found, which comprises 89,66% of the whole plasmid (Fig. 4). From these, 28 are conserved hypothetical proteins and 1 is a hypothetical protein. Comparing the ORFs of the plasmid with other organisms, we observed that 17 (43%) of the ORFs have similarity with ORFs from *Pseudogulbenkiania ferrooxidans*. No tRNAs genes were found. We also searched for homology with bacteriophages and the BLAST analysis did not give any similarity with any phage genomes.

**Table 1:** General features of *pChV1*.

Lenght, bp	44,212
G + C content	65.96%
Total ORFs	39
Percentage of plasmid sequence constituting coding regions	89.66%
Average ORF lenght, bp	1017
Number of conserved hypothetical proteins	28
Number of hypothetical proteins	1

### Plasmid maintenance genes

The plasmid has at least 4 known genes related to plasmid segregation/replication: *parA*, *parB*, *repA* and a gene with RPA domain, involved in plasmid replication initiation (Table 2). *parA* and *parB* encode the ParA and ParB proteins, respectively. These proteins are part of the Type I plasmid-partitioning system and are responsible for ensuring the correct propagation of plasmids to daughter cells throughout cell division^24^. This partitioning system is founded in prophages, plasmids and chromosomes^25^. RepA is a protein related to plasmid replication and is characteristic of P1 plasmids.

**Table 2:** List of ORFs found in pChV1.

ORF Number	Gene	Length	Domains	Best Hit Identity
ORF_01	Chromosome partitioning protein ParA	840	ParA/Soj/Fer4 NifH	93%
ORF_02	Partitioning protein ParB	885	ParB	96%
ORF_03	Plasmid replication protein RepA	951	Plasmid replication initiator protein	49%
ORF_04	Conserved hypothetical protein	477	none	82%
ORF_05	Conserved hypothetical protein/putative bacteriophage lysis protein	1029	COG4623	82%
ORF_06	Conserved hypothetical protein	312	SlyX	97%
ORF_07	Conserved hypothetical protein	756	Cadherin repeat/Ca2+ binding	32%
ORF_08	Conserved hypothetical protein	1422	none	44%
ORF_09	Conserved hypothetical protein	1887	none	60%
ORF_10	Conserved hypothetical protein	363	none	99%
ORF_11	Conserved hypothetical protein	219	none	95%
ORF_12	Conserved hypothetical protein	1167	DUF4157	48%
ORF_13	Toxin	801	RhsA	52%
ORF_14	Conserved hypothetical protein	708	none	63%
ORF_15	Conserved hypothetical protein	1125	none	98%
ORF_16	Conserved Hypothetical protein	126	none	52%
ORF_17	Conserved hypothetical protein	615	Transposase	99%
ORF_18	Hypothetical protein	726	none	ND
ORF_19	Conserved hypothetical protein	891	DUF4255	95%
ORF_20	Conserved hypothetical protein	1854	ATPase AAA domain	72%
ORF_21	Conserved hypothetical protein	2415	none	62%
ORF_22	Conserved hypothetical protein	453	none	78%
ORF_23	Conserved hypothetical protein	3768	DUF342	95%
ORF_24	Conserved hypothetical protein	2439	none	82%
ORF_25	Conserved hypothetical protein	993	ribonuclease e/dihydrolipoamide succyniltransferase	66%
ORF_26	Conserved hypothetical protein	3168	TIGR02243(phage tail-like region)	77%
ORF_27	Conserved hypothetical protein	441	none	90%
ORF_28	Phage baseplate assembly protein W	399	GPW gp25	71%
ORF_29	Conserved hypothetical protein	1593	Phage Base V	59%
ORF_30	Conserved hypothetical protein	729	Phage Tube	41%
ORF_31	Conserved Hypothetical protein	165	none	52%
ORF_32	Conserved hypothetical protein	456	Phage T4 gp19	40%
ORF_33	Phage tail protein	447	Phage T4 gp19	91%
ORF_34	Phage tail sheath protein	1416	COG3497(phage tail sheath protein FI)	63%
ORF_35	DNA invertase	624	mpi/SR ResInv(Recombinase;DNA binding)/HTH Hin like	97%
ORF_36	Conserved hypothetical protein	348	HTH_XRE (transcriptional regulator family)/xenobiotic response	97%
ORF_37	Toxin HipA	1350	HipA/Rna Pol	90%
ORF_38	Conserved Hypothetical protein	126	none	52%
ORF_39	Plasmid replication initiator protein	1182	RPA	54%

### Structural phage genes

An abundant number of genes related to phage structure are present in the sequence of the plasmid. Genes that codify the baseplate, sheath and tail proteins as well as conserved hypothetical genes with domains related to phage structure are in close proximity in the pChV1 sequence.

### Other genes

A DNA invertase (ORF_35), an enzyme that catalyzes site-specific recombination in phages was found. A conserved hypothetical protein (ORF_17) with a transposase domain is also present in the plasmid sequence. Toxins (ORF_13 and ORF_37) that may be related to the toxin-antitoxin (TA) system responsible for assuring the survival only for the cells with a copy of the lisogenyzed phage were also located. Other worthy-mention genes are: conserved hypothetical proteins with Ribonuclease E domain, XRE domain and ATPase AAA domain.

### GC profile and GC-skew

**Figure 5:**
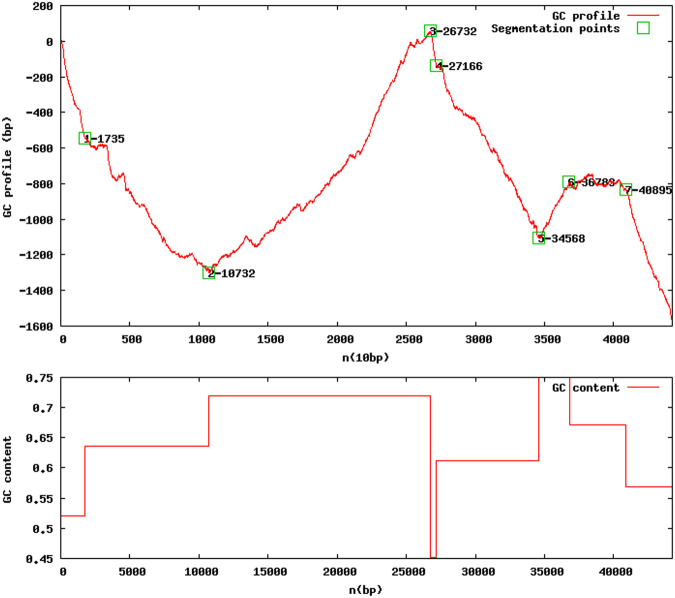
GC content profile of pChV1. Two points of low GC content are observable in the 1) beginning-end of the plasmid sequence and in the 2) 27000 bp region (second chart, below).

**Figure 6:**
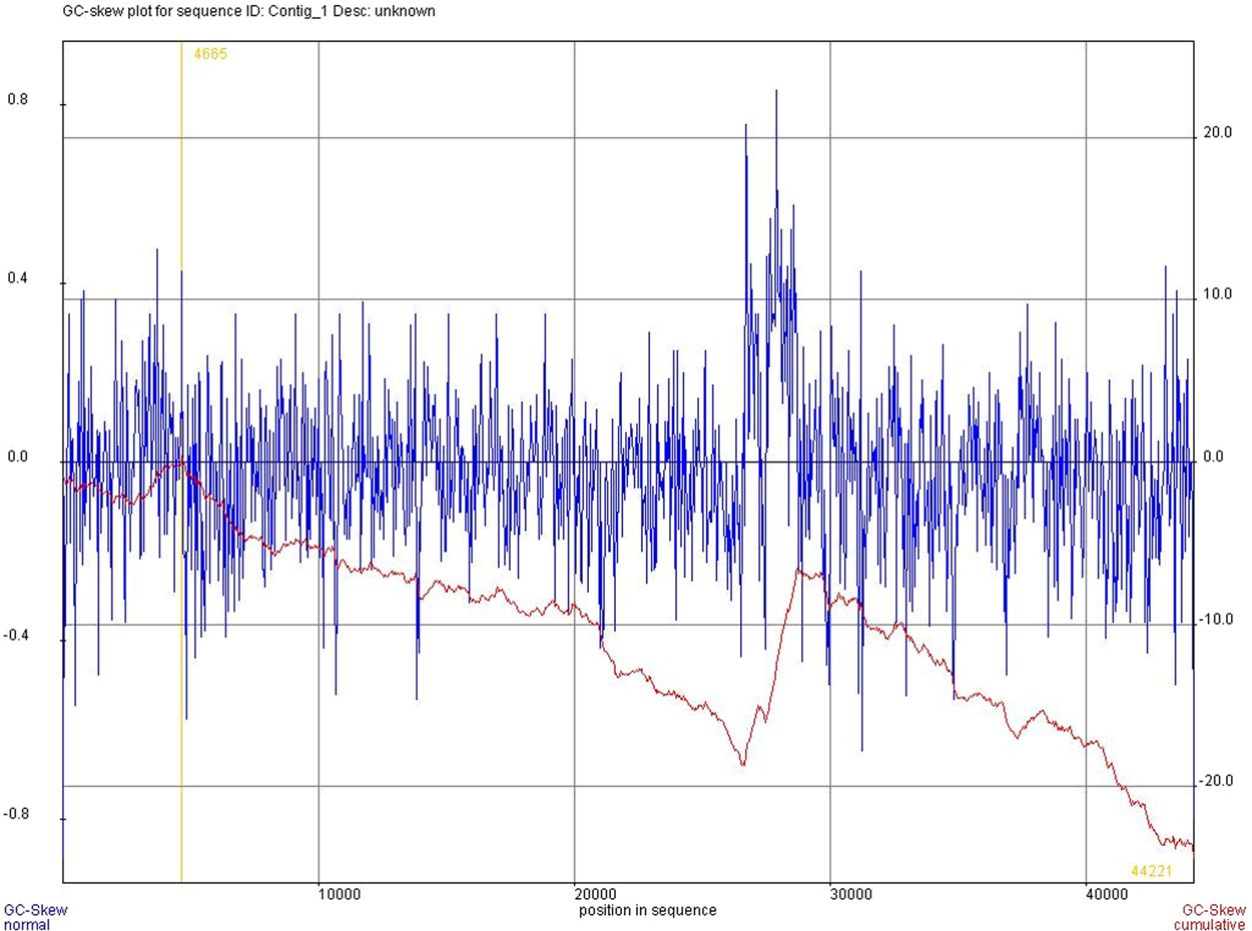
GC-skew of pChV1. The cumulative GC-skew (red curve) has two decline points, characteristic of origins of replication and might reflect *oriR* and *oriL*.

We were able to identify two points in the sequence of pChV1 where the GC content drops when comparing to the whole sequence (Fig. 5). These variations also qualitatively agree with intensity variaitons in the single molecules studies in Fig. 3. This might reflect the presence of two origins of replication that are present in P1-like plasmids, *oriR* and *oriL*. GC-skew also helps predicting the location of the leading and lagging strand and cumulative GC-skew values reflect the origin and terminus points of replication^26^. In our analysis, we can observe throughout the cumulative GC-skew curve, two regions that we could call minimum points that sign the origins of replication *oriR* and *oriL* (Fig. 6).

### Repeated and palindromic sequences

**Figure 7:**
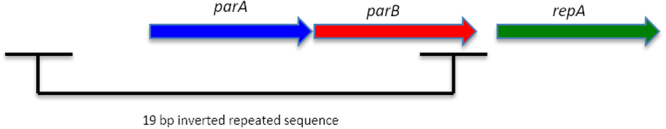
Schematic chart showing the genomic context in which the 19 bp inverted repeated sequence are flanking the partitioning-related genes *parA* and *parB*. The diagram is not to scale.

A 19 bp inverted repeated sequence separated by 1,785 bp was also located and may be involved in the circularization of the phage or other homologous recombination-based process (Table 3). This pair of sequences is located flanking the partitioning related genes *parA* and *parB* (Fig. 7). Other inverted repeat sequences with size varying from 23 to 54 bp were also founded although the complementarity between the pair of repeats was not 100% (data not shown). Palindromic sequences located at two distinct sites in the sequence and varying from 7 to 17 bp are also present (Table 3).

**Table 3:** Pairs of Inverted repeated and palindromic sequences founded in pChV1.

Coordinates	Strand	Sequence	Type of sequence
42355–42373 44177–44159	+ +	TGTAGCAAGTTGCTACACT ACATCGTTCAACGATGTGA	Inverted repeat sequence
5113–5120 10723–10730	+ +	AAATATTT	Palindrome
44161–44177 42355–42371	− +	TGTAGCAAg/cTTGCTACA	Palindrome
27010–27016 26990–26996	− +	ATAt/aTAT	Palindrome
1608–1615 32309–32316	+ +	TGAATTCA	Palindrome
1465–1478 41121–41134	+ −	TTTTTAACTAAAAA TTTATAGTTAAGAA	Palindrome
304–312 132–140	− +	TTGAt/aTCAA	Palindrome
34202–34209 33338–33345	+ −	AATTAATT AAGTAATT	Palindrome
34414–34424 4595–4605	+ −	ATTTGTCATAT	Palindrome
27884–27891 32052–32059	− −	TATTCATA	Palindrome

## Discussion

The first bacteriophage was discovered in the 1950’s^27^ and since then, the number and variety of new viruses that infect bacteria has grown considerably, reaching more than 1,300 genome projects according to the NCBI database. While researching the opportunistic pathogen *C. violaceum*, in genomic preparations we observed an extra-chromosomal DNA of high molecular weight (but lower than it would be if it was genomic DNA). We then isolated and sequenced this putative plasmid which proved to have genes from the P1 bacteriophage/plasmid group.

After the sequencing of *C. violaceum*^7^, the presence of four different sequences of prophages (CvP1-4) were observed in the *C. violaceum*’s genome^28^. Neither of these is related to the plasmid we report here. Before this, tail-like particles were observed in *C*. violaceum by electron microscopy although no biological activity was associated to them^29,30^.

According to the sequence data and annotation, the plasmid founded in *C. violaceum* could be a P1-like virus due to the presence of genes that encode for structural viral particles. Moreover, genes related to plasmid partitioning and the plasmid initiator protein RepA are strong evidence to classify this plasmid as a P1-like phage. Another hallmark of P1-like phages is the presence of toxin-antitoxin genes that constitute a plasmid addiction system. In pChV1 two ORFs are predicted to be toxin genes (ORFs 13 and 37 with 52% and 90% of identity, respectively) although further studies need to be done to confirm the presence of this system.

From our search for homology, we observed that pChV1 has a nucleotide sequence very different from other phages described so far. This feature hampers the search for phage-related sequences, such as *lox* sites, *incC* and *incA* and others, which are, in general, well conserved between other viruses, but does not exclude the existence of them in pChV1. However, repeated sequences that are founded amongst other phages are also present in pChV1, such as the 19 bp inverted repeated sequence (Fig. 7).

Origins of replication are GC-poor regions and locating them in the plasmid may suggest the locals where replication starts. Although we were not able to predict specific sequences that would correspond to origins of replications in pChV1, the GC content profile and GC-skew showed two regions that might reflect *oriR* and *oriL*. *oriR*, that is used during plasmid maintenance replication, is in the same region as the *parA*, *parB* and *repA* genes. This co-location of a possible origin of replication and the plasmid maintenance genes is observable in pChV1. Conversely, *oriL* is related to lytic growth and is separated about 9 kb from *oriR* in P1^31^. We suggest that the second possible origin of replication founded in pChV1 (located approximately at 27 kbp) corresponds to *oriL*.

One notable feature is that when we aligned the predicted open reading frames using BLASTn (that searches a nucleotide query in a nucleotide database) we obtained no significant result. Conversely, when BLASTx was used (searches a translated nucleotide query in a protein database) we were able to identify genes with high degree of confidence. This means that during evolution this virus accumulated many mutations on its DNA sequence but conserved – to some extent - the amino acid composition of its proteins. For example, pChV1 has many ORFs with more than 90% of identity with other genes found in bacteria (Table 2). When we aligned these same ORFs using BLASTn we did not obtain any significant result.

Besides the presence of phage-related genes and sequences, some essential elements that would make pChV1 a functional P1-phage are still missing^31^. By the lack of evidence, we cannot conclude if this plasmid is a temperate P1-like phage, or if it is a chimeric DNA, part bacteriophage or plasmid. Moreover, it could be a fragment of DNA that is maintained inside *C. violaceum* by addiction systems but defective in its capacity of lisogeny. Conversely, the tail-like particles observed in the 1970s^29,30^ could be an evidence that, under stress, the phage proteins encoded by pChV1 would be produced.

Genetic mobile elements are still important in the field of molecular biology. Beside this, the use of phage-derived systems as tools has allowed genome manipulation of all kind of organisms. In this way, further study of pChV1 would bring new ways to investigate genetic aspects of *C. violaceum* and maybe other species. Finally, pChV1 with its great number of hypothetical ORFs, is a rich reservoir of unexplored genes that might contribute to our understanding of the mechanisms underlying viral infections and plasmids.

## Conclusion

In our work, we discovered an extra-chromosomal DNA – that we named pChV1 - in the opportunistic pathogen *Chromobacterium violaceum*. This plasmid is present as a low-copy plasmid and has most of its genetic apparatus composed of ORFs with unknown function, making pChV1 an important source of genes to be further explored. More than this, when its biology is better understood, this element can be used in genetic studies in *C. violaceum* as well as in other organisms.
